# Editorial

**Published:** 1841-12-25

**Authors:** 


### THE MEDICAL EXAMINER.

PHILADELPHIA, DEC. 25, 1841.

On Saturday, December 11th, and again on the 18th, we had the pleasure of witnessing, by invitation, several operations by Professor Pancoast at the Philadelphia Almshouse Hospital. The first was an amputation at the knee joint, performed in a case of necrosis and caries affecting nearly the whole length of the tibia. The ulcerous action was making rapid progress in the cancellated structure of the head of the bone, and cicatrices, the result of ulceration around the orifices in the soft parts which gave exit to the pus, approached so near the knee as to preclude the possibility of forming a healthy flap in amputation through the bones of the leg. The operator, in his address to the class, prior to the introduction of the patient, dwelt at length upon the mortality attendant upon amputation at other points than the joints, especially from phlebitis and metastatic abscess, drawing his conclusions from what have been recently considered as furnishing the least disputable data—the *tabular results* of hospitals and individual surgeons. He argued to a conclusion which we do not esteem an absolutely legitimate one; namely, that amputation of the thigh must necessarily prove more mortal than the average, because it involves so great a mass of important organs; and he decided upon amputation at the knee joint, in consequence of the smaller extent of highly important soft parts interested in the incision. He thought that, fewer veins of importance being implicated, there would be less chance of phlebitis in this operation than in that usually performed above the knee. Moreover, he believed that the stump would be more available, from the great breadth of the bone at this place and the length of the lever remaining, in accomplishing the various motions of the limb. But he admitted that this mode of operating was still, in all particulars, *sub judice*.

We are not fully prepared to join issue with the Professor, at present, on any of these points of opinion. They should have their just weight in such questions, but we are inclined to doubt whether that weight will prove to be considerable. Nothing is more likely to mislead the surgeon (or, indeed, *the physician*, who is not a very accurate reasoner,) than too exclusive dependence upon statistical tables of results. These tables seldom involve the tythe of the data necessary to a wise conclusion upon the propriety or impropriety of any given operation; and still less frequently do they enable us to separate the unfortunate results due to the *nature of the operation* from those which are due *simply to the mere fact of operating at all*, under peculiar endemic or epidemic influences.

Results deduced from a series of observations upon the graver class of operations, made during the prevalence of erysipelas, and presented—jumbled together with a mass of cases collected under other circumstances—in a barren, tabular form, are worse than useless,—*they are absolutely deceptive*—unless employed exclusively by the really philosophical surgeon. A series of positive observations upon any particular accident in the crowded wards and foul atmosphere of the *Hotel Dieu*, should be regarded as of little weight in regulating practice in the free halls of American hospitals. Add to this, that in the first rise of a novel plan of treatment, a dozen happy cases may appear to establish a practice,—as, in the first series of amputations at the knee by M. Velpeau—yet, that half a dozen next succeeding may be so unfortunate as to lead to its repudiation; as, in the second series of observations by the same justly distinguished operator. And it does not even follow, necessarily, that the merits of the operation itself have any essential connection with either the favourable or the unfavourable results; *they may all be* justly attributable to fortuitous circumstances incapable of presentation in a table. Until, then, we can cause the acumen in diagnosis, the moral dependability of operators, the atmospheric and other hygienic circumstances of each patient, the epidemic and endemic peculiarities of the season, the particular constitution, bodily and mental, of the patient, and the condition of the parts through which the incisions have been carried, together with many other points, to be fairly stated, we must confess that we have far more confidence in certain general principles, with a fair proportion of common sense in applying them, than we have in any application of the science of numbers to the profession of medicine. Frankly, we do not believe—the evidence of tables to the contrary notwithstanding—that the amputation of the thigh—*per se*—is very dangerous to life!

But we are wandering from our subject. There can be no doubt that the amputation at the knee joint involves fewer parts of importance than that performed at any other point between the ankle and the hip—and the only questions involved in the discussion of its propriety are the capacity of the synovial and other articular tissues to support the consequences of the operation without engendering dangerous irritation, and the ability of the patient to endure the exhaustion of extensive suppuration from a wide expanse of flap and stump. We felt convinced, before the operation, that union by the first intention, to any very great extent, would be rendered impossible, even between the lips of the flap, by the flow of altered or vitiated synovia from the remnants of the membrane, and from the great rectal bursa; and it will be seen in the sequel that this anticipation was correct. The whole inside of the flap is necessarily suppurating, and the whole surface of the joint, and, doubtless, the bursa also—notwithstanding the singular assertion of M. Velpeau that this sack is closed by the retraction of the patella and thus prevented from complicating the case—are still pouring out synovia.

But, let us describe the operation. The patient was a negress of about middle age. The disease had continued many years, and during the last summer, the exhaustion from the discharge threatened the destruction of life. The invigorating effect of the cold weather brought about a rally of the constitution, but it appeared improbable that the relaxation of another summer could be endured in safety. There was, therefore, no question of the propriety of amputation *somewhere* in this case, seldom as this operation is really warrantable in necrosis. The condition of the skin on the anterior part of the leg limited the length of the flap in front to about the breadth of three fingers from the patella; but laterally and in the rear, there was an ample supply of integuments.

The patient was placed upon the table face downwards, with the legs extended beyond the edge. The tournequette was applied to the popliteal artery very near the knee. An incision was made directly across the limb, as far beneath the attachment of the ligament of the patella as the state of the skin permitted. It was carried around about one-third of the circumference of the limb. From its two extremities, two other incisions were made, sweeping downwards and towards the calf, and then reascending until they met at a very acute angle, just at the lower part of the ham opposite the knee-joint. These incisions included two semi-elliptical flaps of integuments, one on the outer and the other on the inner side of the leg, the long axis of the ellipse being parallel to the long axis of the leg, and the flaps extending nearly three inches below the first or transverse incision. These flaps were detached and reverted, together with the edge of the transverse incision, as high as the patella. The ligament of the patella was then divided, as were also, in due order, the external and internal lateral ligaments. Access to the joint was then perfectly easy. The crucial ligament was detached, and the knee flexed and dislocated. The soft parts of the ham were divided by a stroke or two from the scalpel, (the only cutting instrument used,) and the leg removed. The remains of the lateral ligaments were cut away from the sides of the condyles—the popliteal artery tied, and the tourniquet relaxed—Two or three small vessels then required the ligature. Very little blood was lost, and that little chiefly venous. The flaps were ample, and, after the application of two or three sutures, were bound down to the articular surface by gentle pressure applied by bandage over compresses, and the patient was removed, with a pulse very slightly influenced by the operation.

This morning, Dec. 18th, one week after the operation, we were permitted to examine the stump. Union to the extent of several inches —perhaps six—had taken place posteriorly between the edges of the elliptical flaps. Anteriorly, between those flaps and the edge of the first or transverse incision, granulations were found, but there was no attempt at adhesion. On the removal of the adhesive strips, a slight parting of the edges brought into view the surface of the articular cartilage, smooth, shining, and perfectly unaltered. Free, but not profuse suppuration was going on evidently over the whole interior of the flaps, and a moderate discharge of synovia was mingled with the healthy pus. The patient has not suffered any disturbance of natural rest, or any notable access of fever since the operation. Her countenance displays cheerfulness, and more vigor than it exhibited prior to the operation. There is no inflammatory action observable, as yet, about the stump.

No case could be more promising up to this moment; and we consider it a piece of good fortune to have the opportunity of judging by actual observation, the ulterior consequences of this operation, free from all complications. Both the operator and ourselves were under the impression that this was the first case of amputation of the knee-joint performed in America, but we are informed that it was resorted to by a surgeon in Florida some years ago, and that the stump is a very available one, the patient having since visited Paris to obtain a suitable wooden leg. We should be glad if any of our correspondents could furnish us with the details of the case, or a reference to them if published. In Dr. Pancoast's case, it is evident that the skin which must bear the greatest amount of pressure when the patient has recovered, is that originally supplied from the lateral parts of the leg. This, it appears to us, must also be th e case when circumtances admit of the employment of the circular flap of Velpeau. The oval flap of Baudens is objectionable in every point of view, and was very properly condemned by the Professor in his introductory remarks prior to the operation.

If the operation should become established—which we by no means anticipate—we would suggest the propriety of taking the greater part of the flap from the anterior and lateral parts of the leg, when it can be obtained there, in order to throw the cicatrix altogether behind the prominence of the condyles, which would secure us the advantage of bringing the pressure of the body entirely upon the part of the integuments most capable of enduring it.

Our limits compel us to defer our remarks upon another interesting operation by Dr. P., and some further observations on the case of anchylosis of Professor Gibson, &c., until the appearance of the first number of the new series, in January.

